# A Novel Machine Learning Algorithm Combined With Multivariate Analysis for the Prognosis of Renal Collecting Duct Carcinoma

**DOI:** 10.3389/fonc.2021.777735

**Published:** 2022-01-13

**Authors:** Liwei Wei, Yongdi Huang, Zheng Chen, Jinhua Li, Guangyi Huang, Xiaoping Qin, Lihong Cui, Yumin Zhuo

**Affiliations:** ^1^ Department of Urology, The First Affiliated Hospital of Jinan University, Guangzhou, China; ^2^ College of Mathematics and Physics, Beijing University of Chemical Technology, Beijing, China

**Keywords:** renal cell carcinoma, collecting duct carcinoma, machine learning, predictive model, SEER database

## Abstract

**Objectives:**

To investigate the clinical and non-clinical characteristics that may affect the prognosis of patients with renal collecting duct carcinoma (CDC) and to develop an accurate prognostic model for this disease.

**Methods:**

The characteristics of 215 CDC patients were obtained from the U.S. National Cancer Institute’s surveillance, epidemiology and end results database from 2004 to 2016. Univariate Cox proportional hazard model and Kaplan-Meier analysis were used to compare the impact of different factors on overall survival (OS). 10 variables were included to establish a machine learning (ML) model. Model performance was evaluated by the receiver operating characteristic curves (ROC) and calibration plots for predictive accuracy and decision curve analysis (DCA) were obtained to estimate its clinical benefits.

**Results:**

The median follow-up and survival time was 16 months during which 164 (76.3%) patients died. 4.2, 32.1, 50.7 and 13.0% of patients were histological grade I, II, III, and IV, respectively. At diagnosis up to 61.9% of patients presented with a pT3 stage or higher tumor, and 36.7% of CDC patients had metastatic disease. 10 most clinical and non-clinical factors including M stage, tumor size, T stage, histological grade, N stage, radiotherapy, chemotherapy, age at diagnosis, surgery and the geographical region where the care delivered was either purchased or referred and these were allocated 95, 82, 78, 72, 49, 38, 36, 35, 28 and 21 points, respectively. The points were calculated by the XGBoost according to their importance. The XGBoost models showed the best predictive performance compared with other algorithms. DCA showed our models could be used to support clinical decisions in 1-3-year OS models.

**Conclusions:**

Our ML models had the highest predictive accuracy and net benefits, which may potentially help clinicians to make clinical decisions and follow-up strategies for patients with CDC. Larger studies are needed to better understand this aggressive tumor.

## Introduction

Renal cell carcinoma (RCC) is one of the most common malignant tumors of the genitourinary system. Cancer statistics 2021 reported that about 48780 new cases of RCC in men and 27300 new cases in women will be potentially be diagnosed in the United States during 2021, representing the 6th (5%) and the 9th (3%) most common cancer, respectively (1). Collecting duct carcinoma (CDC), which arises from the principal cells of distal segment of the collecting ducts of Bellini in the renal medulla, is a highly aggressive subtype of RCC (2). CDC is rare in clinical practice and it accounts for 1 to 2% of all renal tumors. Because of its highly malignant biological behavior and most patients are usually diagnosed with local progressive or even distant metastatic diseases, the prognosis is very poor, with a median survival ranging from 10 to 13 months (3–5). Several studies have reported on small samples of CDC cases and investigated the their clinico-pathological characteristics and prognosis (6, 7). However, these studies did not have a large sample size and did not include some non-clinical factors, such as race, marital status and economic status, which have been shown to be risk factors in other researches (8–10). In addition, there is no model to predict the prognosis of CDC, and an accurate prediction model is important for patient follow-up and treatment decisions (11).

Machine Learning (ML) is an emerging intersection approach that involves several disciplines, which is good at associating multiple discrete variables and accurately predicting outcomes. With the rapid growth of evidence-based medicine as well as vast and complicated medical data acquisition, a more advanced tool is required. ML is emerging as a viable alternative for illness diagnosis and prognostic prediction, with a high predictive performance and a wide range of applications (12, 13).

With these considerations, we utilized data from the Surveillance, Epidemiology, and End Results (SEER) database of CDC patients for analysis. This is a publicly available database established by the National Cancer Institute that contains de-identified cancer patients, thus providing a platform for cancer research. Our study aimed to use ML methods to analyze the role of clinical and non-clinical factors on prognosis. In addition, we established a ML model to predict the overall survival (OS) rate of CDC patients.

## Material and Methods

### Data Acquisition and Study Population

This study used the SEER database (https://seer.cancer.gov/) from the National Cancer Institute, which is a freely available cancer registry in the United States. We were granted access to the SEER database files, and all authors adhered to the SEER database’s rules throughout the study. Individual informed consents were not required because no personally identifiable information was used in this study. This work was examined and approved by the Jinan University’s First Affiliated Hospital’s Medical Ethics Committee.

Patients’ data were obtained from the SEER 18 Regs Custom Data (with additional treatment fields), Nov 2018 Sub (1975-2016 variation) by using the SEER*Stat 8.3.9.1 software. The selection criteria included patients diagnosed with histologically confirmed CDC of the kidney (site code C64.9-Kidney, Histologic type ICD-O-3 8319/3). Patients were diagnosed between 2004 and 2016, and those who did not undergo surgery or who underwent only partial nephrectomy (PN) and radical nephrectomy (RN) were included. The exclusion criteria in this study were as follows: (a) unknown American Joint Committee (AJCC) on Cancer 6th TNM stage; (b) unknown marital status; (c) unknown race; (d) unknown purchased/referred care delivery area (PRCDA); (e) regional median family income; (f) unknown laterality of the tumor; (g) unknown tumor size; (h) unknown histological grade; (j) unknown radiotherapy and chemotherapy records and (k) unknown survival months. The downloaded patient data included 243 cases, and the cases who were alive but had survival times of less than 36 months at the follow-up cut-of date were excluded. 215 patients remained in the final cohort and were used in model development.

### Variable Selection and Endpoints

In order to take full advantage of the ML algorithm, multiple readily available clinical and non-clinical characteristics were chosen as independent variables for analysis. Several clinico-pathological variables that are commonly used in cancer research were selected, including AJCC TNM stage, tumor size, laterality of the tumor, histological grade and surgical method used as well as radiotherapy and chemotherapy records. In addition, the non-clinical variables included age at diagnosis, sex, race, marital status, PRCDA and regional median family income. PRCDA is a geographic area within which either the purchased or referred care is made available by the health service to members who reside within the area and can be used as a rough guide to determine where the patients live. Regional median family income was used to broadly assess the economic status of patients. The endpoints of our predictive models were the 1-, 2- and 3-year OS.

### Statistical Analysis and Model Establishment

SPSS (version 22, IBM SPSS software Foundation) and Python (version 3.8.1, Python Software Foundation) was used for all statistical analyses. Firstly, we used a Mann-Whitney U test and a chi-square test to compare continuous and categorical variables, respectively, to assess the distribution of baseline characteristics. For continuous variables, the data were presented as means ± standard deviations (SDs) and for categorical variables, as frequencies (%). Secondly, we calculated the importance of each variable using the XGBoost (XGB) algorithm and assigned weight values to them, with higher scores indicating more importance for the prediction target. XGB is an optimized distributed gradient boosting program and it implements ML algorithms under the Gradient Boosting framework. The univariate Cox proportional hazard model was used to compare the impact of different factors on OS, and then the risk factors were compared with the most important variables that were calculated by the XGB algorithm. Then we used Kaplan-Meier survival estimate to compare survival of patients for each variable. Thirdly, all the patients studied were randomly divided into a training and test set at a ratio of 7:3. The chi-square test was used to analyze the differences between the training and test sets. Several variables with the greatest impact on survival were used for model construction. The training set was used to establish the XGB model and the test set was applied to evaluate it, and we first used 600 trees in XGB in order to build the model.

### Model Improvement and Predictive Performance

We analyzed the results of feature importance and risk factors and then selected the following characteristics for model establishment: TNM stage, tumor size, histological grade, age at diagnosis, region and three types of treatment methods. These characteristics were significant in the survival analyses. To ensure that the model was stable, a ten-fold cross-validation was adopted to evaluate its predictive capability. Our model was then tested and adjusted repeatedly, and the parameters were confirmed for the best model obtained.

Performance analysis comprised of three components. Firstly, model discrimination was quantified with receiver operating characteristic (ROC) curve analysis, and its predictive accuracy was assessed using the area under the ROC curve (AUC). Secondly, we used calibration plots to evaluate the performance of the model, which indicated the calibration and how far the predictions of model deviated from the actual event. Thirdly, clinical usefulness and net benefits were assessed with DCA, which could estimate the net benefit of a model by the calculating the difference between the true- and false-positive rates and weighted these by the odds of the selected threshold probability of risks involved. Also, additional ML algorithms such as multivariate logistic regression (MLR) and classifier (Ada) as well as Naive Bayes classification (NBC) were introduced for comparison. ROC curves and calibration plots were used to further evaluate the appropriateness and generalizability of our model.

## Results

### Baseline Characteristics

A total of 215 CDC patients were included in our study and their clinico-pathological characteristics are provided in **Table 1**. All patients were diagnosed after histological confirmation. The patients had a mean age of 60.6 (median, 61 years; range, 14–89) years. 147 (68.4%) patients were males and 68 (31.6%) were females. A total of 155 (72.1%) were American White, 50 (23.3%) were African American and 10 (4.7%) were Asian or Pacific Islanders. The majority of patients were married (65.1%). There were 86 (40%), 29 (13.5%), 92 (42.8%) and 8 (3.7%) patients from the Eastern United States, Northern Plains, Pacific Coast and Southwestern United States, respectively. Patients with histological grade I, II, III and IV accounted for 4.2% (n = 9), 32.1% (n = 69), 50.7% (n = 109) and 13.0% (n = 28) of all patients, respectively. At the time of diagnosis up to 61.9% of patients presented with a pT3 stage or higher tumor, and 36.7% of CDC patients had metastatic disease. Among all included patients, PN was performed in 13 (6.1%) patients, RN in 185 (86.0%) patients and 17 (7.9%) patients did not undergo surgery. Other details of the baseline information are tabulated in **Table 1**.

**Table 1:** The baseline characteristics of the CDC patients from this study.

Characteristic	Total	Training set	Test set	*p* value
**Total**	215 (100.0%)	150 (70.0%)	65 (30.0%)	
**Survival month**	37.06 ± 42.97	35.33 ± 42.94	41.05 ± 43.01	0.489
**Age at diagnosis**				0.241
**≥65**	82 (38.1%)	61 (40.7%)	21 (32.3%)	
**<65**	133 (61.9%)	87 (59.3%)	44 (67.7%)	
**Race**				0.338
** Black**	50 (23.3%)	31 (20.7%)	19 (29.2%)	
** Other**	10 (5.4%)	9 (6.0%)	1 (1.6%)	
** White**	165 (71.3%)	110 (73.3%)	45 (69.2%)	
**Marital status**				0.683
** Married**	182 (84.7%)	128 (85.3%)	54 (83.1%)	
** Unmarried**	33 (15.3%)	22 (14.7%)	11 (16.9%)	
**Sex**				0.859
** Female**	68 (31.6%)	48 (32.0%)	20 (30.7%)	
** Male**	167 (68.4%)	102 (68.0%)	45 (69.2%)	
**Region**				0.248
** East**	86 (40.0%)	56 (37.3%)	30 (46.1%)	
** Northern Plains**	29 (13.5%)	22 (14.7%)	7 (10.8%)	
** Pacific Coast**	92 (42.8%)	65 (43.3%)	27 (41.5%)	
** Southwest**	8 (3.7%)	7 (4.7%)	1 (1.6%)	
**Income**				0.881
** Low**	13 (6.0%)	8 (5.3%)	5 (7.7%)	
** Middle**	174 (80.9%)	124 (82.7%)	50 (76.9%)	
** High**	28 (13.1%)	18 (12.0%)	10 (15.4%)	
**Laterality**				0.567
** Left**	116 (54.0%)	79 (52.7%)	37 (56.9%)	
** Right**	99 (46.0%)	71 (47.3%)	28 (43.1%)	
**T stage**				0.649
** T1**	69 (32.1%)	49 (32.7%)	20 (30.8%)	
** T2**	13 (6.0%)	6 (4.0%)	7 (10.8%)	
** T3**	114 (53.0%)	80 (53.3%)	34 (52.3%)	
** T4**	19 (8.9%)	15 (10.0%)	4 (6.1%)	
**N stage**				0.187
** N0**	131 (60.9%)	96 (64.0%)	35 (53.8%)	
** N1**	46 (21.4%)	30 (20.0%)	16 (24.6%)	
** N2**	38 (17.7%)	24 (16.0%)	14 (21.6%)	
**M stage**				0.971
** M0**	136 (63.3%)	95 (63.3%)	41 (63.1%)	
** M1**	79 (36.7%)	55 (36.7%)	24 (36.9%)	
**Tumor Size**				0.684
** 0~40 mm**	59 (27.4%)	38 (25.3%)	21 (32.3%)	
** 40~70 mm**	80 (37.2%)	61 (40.7%)	19 (29.2%)	
** 70~100 mm**	51 (23.7%)	32 (21.3%)	19 (29.2%)	
** >100 mm**	25 (11.7%)	19 (12.7%)	6 (9.3%)	
**Histological grade**				0.732
** Grade I**	9 (4.2%)	4 (2.7%)	5 (7.7%)	
** Grade II**	28 (13.0%)	20 (13.3%)	8 (12.3%)	
** Grade III**	119 (55.3%)	80 (53.3%)	29 (44.6%)	
** Grade IV**	69 (27.5%)	46 (30.7%)	23 (35.4%)	
**Surgery**				0.956
** No**	17 (7.9%)	13 (8.7%)	4 (6.2%)	
** PN**	13 (6.0%)	7 (4.7%)	6 (9.2%)	
** RN**	185 (86.1%)	130 (86.6%)	55 (84.6%)	
**Radiation**				0.532
** Yes**	22 (10.2%)	14 (9.3%)	8 (12.3%)	
** No/unknown**	193 (89.8%)	136 (90.7%)	57 (87.7%)	
**Chemotherapy**				0.885
** Yes**	61 (28.4%)	43 (28.7%)	18 (27.7%)	
** No/unknown**	154 (71.6%)	107 (71.3%)	47 (72.3%)	

Continuous variables are shown as means ± standard deviations; classification-variables are shown as numbers and percentages.

### Important Variables and Survival Analysis

**Figure 1:**
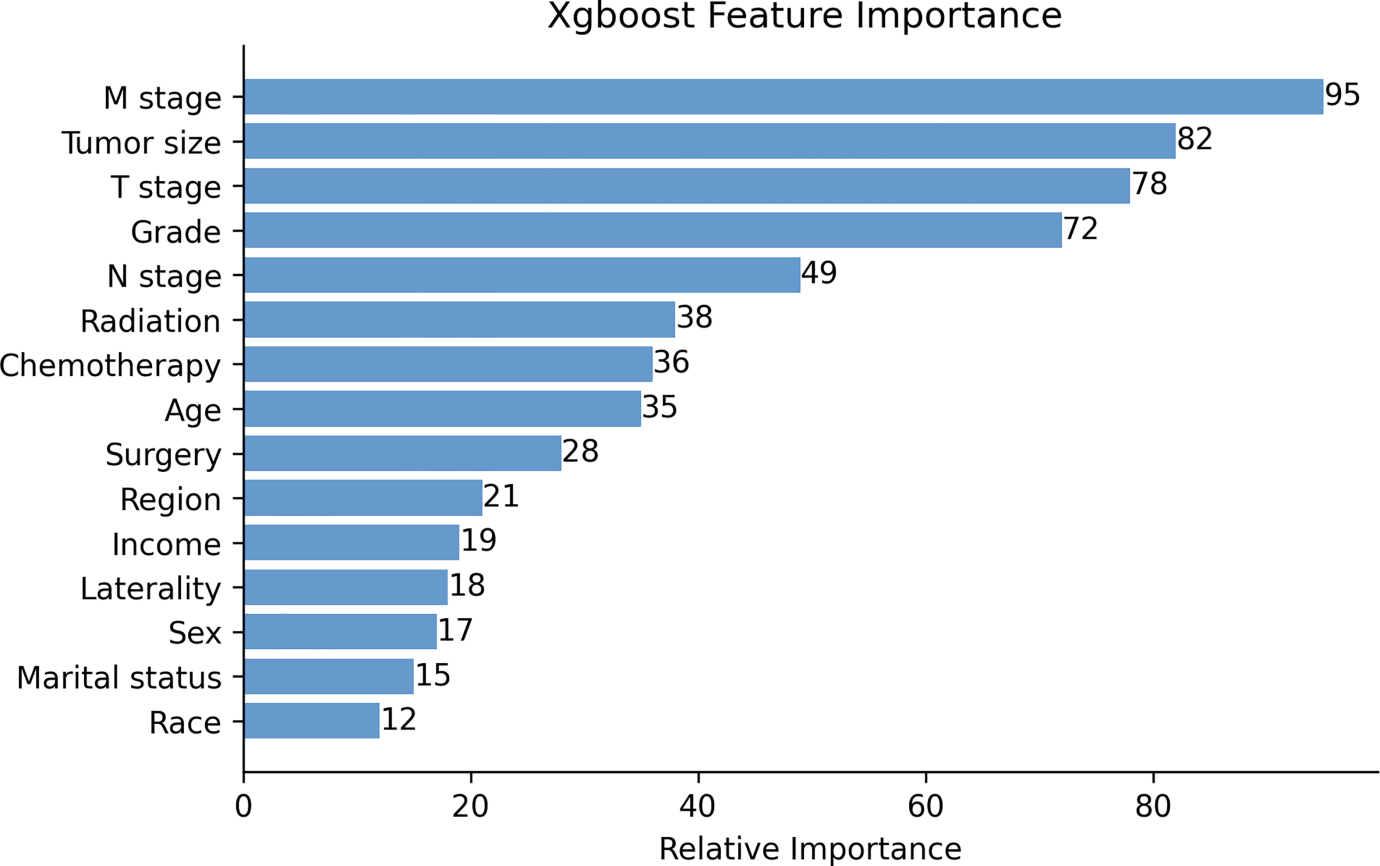
The XGB model was used to calculate the importance of each feature. The bar chart depicts the relative significance of the variables. Region refers to the purchased/referred care delivery area.

The XGB algorithm was used to identify the feature of importance as judged by the size of the gain value obtained for each variable, with the higher values indicating greater importance for the prediction target: M stage (95 points), tumor size (82 points), T stage (78 points), histological grade (72 points), N stage (88 points), radiotherapy (38 points), chemotherapy (36 points), age at diagnosis (35 points), surgery (28 points), the geographical region where the care delivered was either purchased or referred (21 points), income (19 points), laterality (18 points), sex (17 points), marital status (15 points) and race (12 points) (**Figure 1**). With the univariate Cox regression analysis, several high value clinico-pathological variables were defined as risk factors, including age at diagnosis (p=0.002), T stage (p<0.001), N stage (p<0.001), M stage (p<0.001), tumor size (p<0.001), histological grade (p<0.001), surgery (p<0.001), radiotherapy (p<0.001) and chemotherapy (p=0.003). These were also some of the highest scoring variables with respect to the featured importance points allocated. In addition, the univariate Cox regression analysis defined a non-clinical variable, PRCDA, as a risk factor and this was the most important non-clinical feature as calculated by the XGB algorithm (p=0.045) (**Table 2**).

**Table 2:** Kaplan–Meier analysis and univariate Cox regression of overall survival (OS) for collecting duct carcinoma (CDC) patients.

Characteristic	1-year OS %	2-year OS %	3-year OS %	Kaplan-Meier		COX	
				Log Rank χ2 test	*p* value	HR (95% CI)	*p* value
**All**	56.7	41.4	33.0				
**Age at diagnosis**				10.275	0.001		
** Young**	59.3	45.8	39.8			Reference	
** Older**	52.4	34.1	21.9			1.636 (1.201-2.228)	0.002
**Race**				0.760	0.683		
** Black**	60.0	44.0	34.0			Reference	
** Other**	50.0	30.0	30.0			1.367 (0.660-2.832)	0.400
** White**	56.7	41.2	32.9			1.035 (0.718-1.493)	0.853
**Marital status**				1.125	0.288		
** Unmarried**	66.6	51.5	39.3			Reference	
** Married**	54.9	39.5	31.8			1.270 (0.810-1.990)	0.298
**Sex**				0.053	0.816		
** Female**	54.4	42.6	33.8			Reference	
** Male**	57.8	40.8	32.6			0.963 (0.693-1.336)	0.820
**Region**				5.154	0.161		
** East**	52.3	38.3	29.1			Reference	
** Northern Plains**	72.4	62.1	58.6			0.586 (0.348-0.989)	0.045
** Pacific Coast**	54.3	36.9	28.2			1.028(0.738-1.432)	0.871
** Southwest**	75.0	50.0	37.5			0.825 (0.358-1.903)	0.652
**Income**				0.801	0.669		
** Low**	53.8	38.4	30.7			Reference	
** Middle**	55.7	40.8	32.2			0.964 (0.507-1.833)	0.911
** High**	64.2	46.4	39.3			0.781 (0.363-1.680)	0.527
**Laterality**				0.301	0.582		
** Left**	56.0	42.2	35.3			Reference	
** Right**	57.5	40.4	30.3			1.088 (0.801-1.479)	0.589
**T stage**				40.026	<0.001		
** T1**	78.2	66.7	62.3			Reference	
** T2**	69.2	53.8	46.1			1.571 (0.759-3.254)	0.224
** T3**	46.4	29.8	18.4			2.633 (1.796-3.860)	<0.001
** T4**	31.5	10.5	5.2			4.581 (2.600-8.073)	<0.001
**N stage**				41.131	<0.001		
** N0**	70.2	55.7	47.3			Reference	
** N1**	30.4	15.2	10.8			2.871 (1.974-4.176)	<0.001
** N2**	42.1	23.7	10.5			2.396 (1.602-3.585)	<0.001
**M stage**				81.492	<0.001		
** M0**	77.2	56.6	47.7			Reference	
** M1**	21.5	15.1	7.6			3.946 (2.856-5.453)	<0.001
**Tumor Size**				32.817	<0.001		
** 0~40 mm**	76.2	69.4	64.4			Reference	
** 40~70 mm**	58.75	37.5	25.0			2.447 (1.585-3.777)	<0.001
** 70~100 mm**	39.2	23.5	15.6			3.372 (2.106-5.398)	<0.001
** ＞100 mm**	40.0	24.0	20.0			3.283 (1.874-5.750)	<0.001
**Histological grade**				18.052	<0.001		
** Grade I**	77.8	77.8	77.8			Reference	
** Grade II**	78.6	75.0	42.8			1.702 (0.496-5.840)	0.398
** Grade III**	51.3	33.9	22.0			3.695 (1.166-11.708)	0.026
** Grade IV**	53.6	34.7	13.0			4.404 (1.377-14.087)	0.012
**Surgery**				62.639	<0.001		
** No**	5.8	0	0			Reference	
** PN**	100.0	92.3	92.3			0.041 (0.013-0.125)	<0.001
** RN**	58.3	41.6	31.8			0.186 (0.108-0.321)	<0.001
**Radiation**				15.381	<0.001		
** No/unknown**	59.5	44.5	36.7			Reference	
** Yes**	31.8	13.6	0			2.397 (1.514-3.795)	<0.001
**Chemotherapy**				8.966	0.003		
** No/unknown**	61.0	46.7	38.3			Reference	
** Yes**	45.9	27.8	19.6			1.635 (1.175-2.275)	0.004

**Figure 2:**
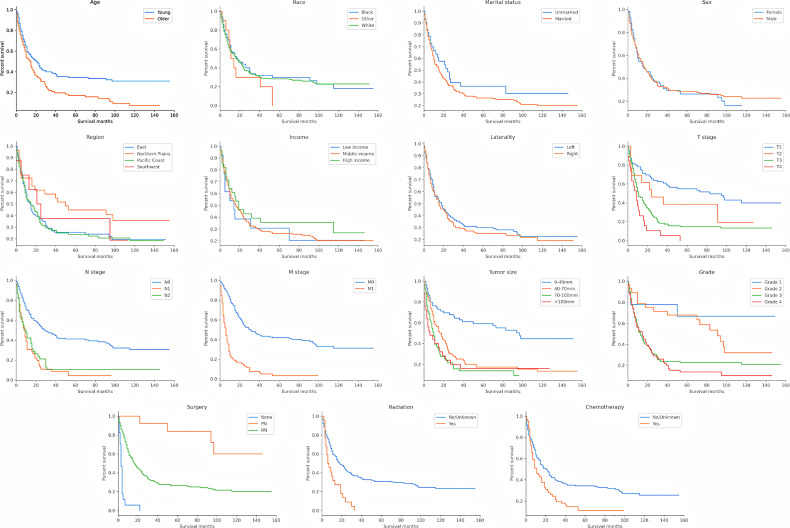
Kaplan–Meier survival curves to evaluate the influence of the all the classified characteristics for CDC patient survival.

According to the Kaplan–Meier survival curves and log-rank tests for categorical variables, T stage, N stage, M stage, tumor size, histological grade, age at diagnosis, radiotherapy, chemotherapy and surgery significantly affected patients’ survival. However, race, marital status, sex, laterality, region and income were not significant factors which influenced OS (**Figure 2** and **Table 2**).

### Model Performance

**Figure 3:**
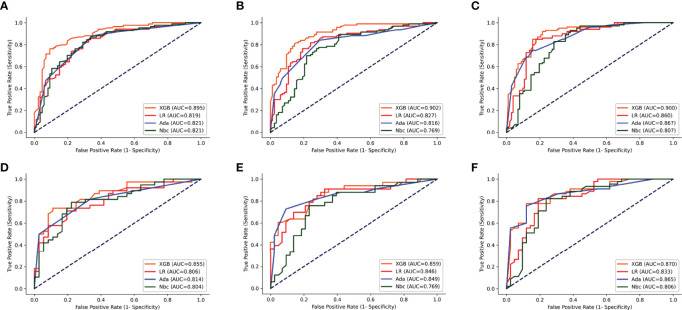
ROC curves of the four models: XGB, Ada, Nbc, and MLR. **(A–C)** training sets of 1-, 2- and 3-year OS predictive model, respectively. **(D–F)** test sets of 1-, 2- and 3-year OS predictive model, respectively. XGBoost, extreme gradient boosting; Ada, Adaptive Boosting; Nbc, Naive Bayes classifier; MLR, multivariate logistic regression.

The parameters for the best model performance were confirmed after several tests and adjustments. To determine the accuracy of our models, the ROC curves and calibration plots for the training set (n = 150) and the test set (n = 65) were constructed. The XGB model had the best performance in the training set of 1-, 2- and 3-year OS (AUC=0.895, 0.902 and 0.900, respectively), compared with MLR (AUC=0.819, 0.827 and 0.860, respectively), Ada (AUC=0.821, 0.816 and 0.867, respectively) and NBC (AUC=0.821, 0.769 and 0.807, respectively) (**Figures 3A–C**). With the test set of 1-, 2- and 3-year OS, the accuracy of the XGB model was also higher (AUC=0.855, 0.859 and 0.870, respectively) than all the other three models (**Figures 3D–F**).

**Figure 4:**
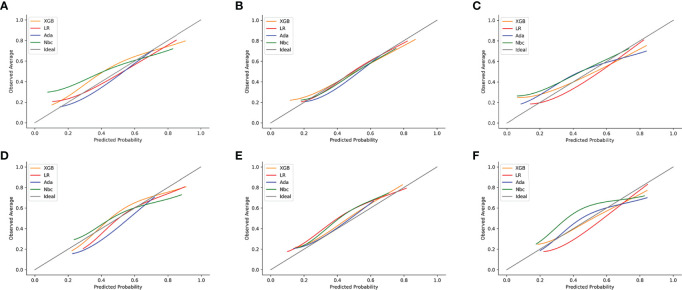
Examples of calibration plots for predicting 1-, 2- and 3-year OS with various models: XGB, Ada, Nbc and MLR. **(A–C)** training sets of 1-, 2- and 3-year OS predictive model, respectively. **(D–F)** test sets of 1-, 2- and 3-year OS predictive model, respectively. The 45-degree straight line on each graph represents the perfect match between the observed (y-axis) and predicted (x-axis) survival probabilities. A closer distance between two curves indicates a greater accuracy.

The calibration plots for the training and test sets of 1-, 2- and 3-year OS indicated that the predictive probabilities against observed survival rates showed excellent concordance in the XGB, followed by the Ada and NBC models, respectively. All the models’ calibration plots of 1-, 2- and 3-year OS were found to be around the 45-degree ideal line. However, the calibration of the traditional MLR model tended to overestimate the risk across the entire range of predicted probabilities when compared with all the other models (**Figure 4**).

**Figure 5:**
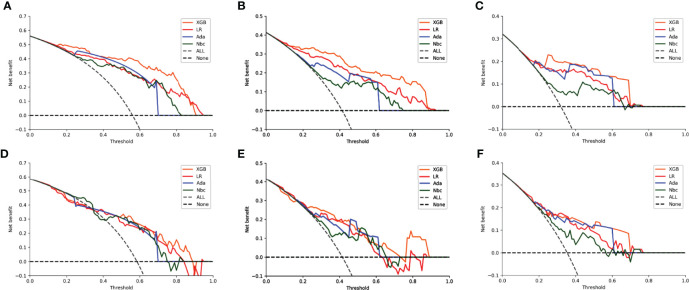
Decision curve analysis graphs showing the net benefit against threshold probabilities based on decisions from model outputs. Curves were obtained based on predictions of the four different models, and the two curves obtained were based on two types of extreme decisions. The curves referred to as ‘All’ represent the prediction that all the patients would die in 1, 2 and 3 years, and the curve referred to as ‘None’ represent the prediction that all the patients would be alive in 1, 2 and 3 years. **(A–C)** training sets of 1-, 2- and 3-year OS predictive model, respectively. **(D–F)** test sets of 1-, 2- and 3-year OS predictive model, respectively.

DCA of the four algorithms were subsequently constructed in our study (**Figure 5**). The y-axis of the decision curve represents the net benefit which is a decision-analytic measure for judging whether any particular clinical decision results in more benefit than harm. Each point on the x-axis represents a threshold probability that differentiates between dead and live patients. This analysis shows that all the models achieved net clinical benefit.

## Discussion

CDC is an invasive tumor with aggressive malignancy and very poor prognosis. As the number of nephrectomy procedures increases each year, the detection rate of CDC may rise (14). The American Society of Anesthesiology (ASA) evaluated the prognosis of CDC based on ASA scores, tumor size, distant metastasis, histological grade and lymphovascular invasion and classified it into three risk groups. The 5-year survival rates were 96, 62 and 8% for the low-, medium- and high-risk groups, respectively (15). Because of the rarity of this tumor subtype, its aggressive behavior (advanced stage at presentation and poor prognosis) and the lack of consistent underlying molecular abnormalities, evaluating new treatment options and reaching consensus standards for management has been a difficult task. Several studies have reported cases of CDC treated with chemotherapy or targeted drugs; for example, Tetsuya reported a relatively good prognosis for individual patients with advanced metastatic CDC after receiving combination immunotherapy treatment (16). However, these studies had only a few cases and did not always stratify patients and they lacked prediction of prognosis of the disease (17–19).

As a new type of artificial intelligence algorithm, XGB is efficient and easy to use, and it can deliver high performance and accuracy when compared to other algorithms. It is an improvement over the gradient boosting decision tree (GBDT), which has significant advantages in preventing overfitting, parallel processing, cross-validation and handling of missing values (20). This novel ML algorithm is becoming increasingly popular in the medical arena and it is widely used for disease prediction and early diagnosis (21, 22). Based on this consideration, we analyzed the clinico-pathological characteristics of CDC patients, and we established a model to predict their prognosis using a novel XGB algorithm as well as combining clinical and non-clinical variables. To our knowledge, this is the first cohort study based on a relatively large number of CDC patients, reporting several clinical and non-clinical characteristics and factors that may affect the prognosis of CDC patients, which has significant implications for clinicians to deepen their understanding of this disease.

In our study, 164 (76.3%) patients died during follow-up, with a median survival time of 16 months, of which 76 patients were diagnosed with distant metastatic disease. Our cohort had a lower metastatic rate and better prognosis than those reported by other research groups (23, 24). In the Kaplan–Meier survival analysis, nine of the 15 selected variables were defined as factors that could affect patient survival, including age at diagnosis, T stage, N stage, M stage, tumor size, histological grade, surgery, radiotherapy and chemotherapy. Surgery could significantly improve the prognosis of patients and those who underwent PN had a better prognosis compared to those who underwent RN. This is probably because only patients with small renal mass and early-stage tumors would accept PN. On the contrary, patients who underwent RN usually had a more advanced stage of the disease and most patients who were inoperable had metastatic disease. Patients who accepted radiotherapy and chemotherapy also had poor prognosis for the same reasons. Physicians may use systemic therapy for those patients with advanced metastases or radiotherapy of the metastatic sites. However, CDC is usually insensitive to these types of treatments (23, 25, 26). In previous studies, marital and economic statuses were reported to be associated with prognosis, but these were not defined as risk factors in the present study (27, 28). This may be due to the extremely poor prognosis of this disease, with a large number of patients dying in a short time.

As expected, we found a high degree of agreement between the most important variables calculated by the XGB model and those variables defined as risk factors in the Cox regression analysis. Nine clinico-pathological characteristics were defined as risk factors by univariate Cox regression and were the highest scoring factors as judged by their importance, and they were also found to have significant effects on patient survival. In addition, several studies have reported the impact of non-clinical factors on disease prognosis (29, 30). In our study, we found the PRCDA was defined as a risk factor in univariate Cox regression. This parameter was also the highest scoring non-clinical variable and the other non-clinical factors were not statistically significant, although a larger cohort of patients may show to differences. Our novel XGB algorithm was able to assign a value to each variable, including non-clinical ones. This model appears to be better at handling multiple discrete variables, which is difficult to achieve with more traditional methods.

In this study, we selected the nine most important clinical variables as well as a non-clinical variable in order to construct three ML models for the 1-, 2- and 3-year OS of CDC patients. Then we used ROC, calibration plots and DCA to evaluate the performance of our models. The predictive accuracy of our XGB models over three different periods was the highest in both training and test sets (1-year OS AUC=0.895 and 0.855, 2-year OS AUC=0.902 and 0.859, 3-year OS AUC=0.900 and 0.870, respectively). Compared to this, the other ML and MLR models were less accurate. These findings suggest that the MLR and other ML models had weaknesses in their accuracy when analyzing the connection between multiple datasets, whereas the XGB model excelled at properly predicting outcomes from numerous unrelated datasets.

We further evaluated the accuracy of the models with calibration plots and similar results were obtained from these analyses when using the three different periods in both the training and test sets. They predicted probabilities against the observed average survival rates indicating that the XGB model had excellent consistency when compared to other models. In addition, the XGB curves obtained here were closer to the ideal lines. The MLR model tended to overestimate the mortality risks across the entire range of predicted probabilities, which means that this might result in a higher false-positive rate and can lead to over-follow up and overtreatment of some patients.

DCA is a novel approach created by the Memorial Sloan Kettering Cancer Center for assessing the efficacy of therapeutic decisions, which takes into account that clinicians often focus on different goals in different situations during clinical practice. The approach uses the net benefits at different thresholds in order to evaluate the clinical utility of a model (31). In our study, whether in the 1-, 2- or 3-year OS model, within the threshold of most ranges, the net benefit of our XGB model was higher than the other models, which indicates that the value of benefits minus the drawbacks is biggest. The results of the DCA illustrated that the benefits and drawbacks of clinical decisions could be well weighed by our model, with overtreatment and frequent follow-ups being largely avoided. They also suggest a more accurate identification of high-risk patients.

Despite several strengths, this study has certain limitations. Firstly, the data from this study are retrospective and prospective clinical data are needed to provide more reliable evidence for the clinical application of the model. Secondly, the model is based on the SEER database which only contains data from the North American population. There may be gaps in population applicability thereby necessitating the study of a broader population base in future research. Thirdly, due to the limited number of cases in the study cohort, it is possible that some variables did not have sufficient power to show statistical significance.

In summary, this study analyzed the clinico-pathological characteristics and prognosis of CDC patients and used a novel artificial intelligence algorithm to construct three prognostic models. Our model performed very well in predicting the prognosis of CDC patients, and could potentially help clinicians to make more accurate and personalized clinical decisions. The practical use of this model will be to help clinicians predict the OS of CDC patients and so deciding potential follow up strategies. For patients, it could make some who are at high-risk, more vigilant, which may improve their long-term prognosis.

## Conclusions

Our study illustrated the clinico-pathological characteristics of CDC patients and nine clinical and a non-clinical variables were defined as risk factors. Our ML models had the highest predictive accuracy and this may potentially help clinicians to make clinical decisions and follow-up strategies for individual patients with CDC. This study will encourage larger studies to be performed so that we can better understand this aggressive tumor.

## Data Availability Statement

The datasets presented in this study can be found in online repositories. The names of the repository/repositories and accession number(s) can be found in the article/**Supplementary Material**.

## Ethics Statement

The studies involving human participants were reviewed and approved by Medical Ethics Committee of Jinan University’s First Affiliated Hospital. Written informed consent from the participants’ legal guardian/next of kin was not required to participate in this study in accordance with the national legislation and the institutional requirements.

## Author Contributions

LW and ZC conceived and designed the study. YZ and LC provided administrative support. LW, GH, and XQ collected and assembled the data. YH, LW, JL, and LC contributed to data processing, interpretation of results, and drafting. All authors read and approved the manuscript.

## Funding

This study was supported by the Leading Specialist Construction Project-Department of Urology, the First Affiliated Hospital, Jinan University (711006). Postdoctoral Fund of the First Affiliated Hospital, Jinan University (809011). Postdoctoral Program of the International Training Program for Outstanding Scientific Research of Guangdong Province (2019). Science and Technology Planning Project of Guangdong (2020A1414010348).

## Conflict of Interest

The authors declare that the research was conducted in the absence of any commercial or financial relationships that could be construed as a potential conflict of interest.

## Publisher’s Note

All claims expressed in this article are solely those of the authors and do not necessarily represent those of their affiliated organizations, or those of the publisher, the editors and the reviewers. Any product that may be evaluated in this article, or claim that may be made by its manufacturer, is not guaranteed or endorsed by the publisher.
